# Diagnostic pitfalls in IgG4-related sclerosing cholangitis presenting as perihilar cholangiocarcinoma: case report with literature review

**DOI:** 10.3389/fimmu.2026.1684445

**Published:** 2026-04-17

**Authors:** Yu Yang, Yiwei Hou, Yashan Wang, Qingjin Wan, Yunxi Fu, Meiqi Jing, Li Yi, Xiaolu Wang, Mingzheng Hu, Rongchun Xing

**Affiliations:** 1 The First College of Clinical Medical Science, China Three Gorges University, Yichang, Hubei, China; 2 Department of Hepatobiliary Surgery, Yichang Central People’s Hospital, Yichang, Hubei, China; 3 Department of Endocrinology, Yichang Central People’s Hospital, Yichang, Hubei, China; 4 Medical Technology College of Qiqihar Medical College, Qiqihar, Heilongjiang, China; 5 Department of Nuclear Medicine, The Affiliated Hospital of Southwest Medical University, Luzhou, Sichuan, China; 6 Department of Pathology, Yichang Central People’s Hospital, Yichang, Hubei, China

**Keywords:** autoimmune biliary disorder, corticosteroid therapy, histopathological diagnosis, IgG4-related sclerosing cholangitis, mimic of perihilar cholangiocarcinoma

## Abstract

**Background:**

IgG4-related sclerosing cholangitis (IgG4-SC) is an uncommon autoimmune biliary disorder that often closely mimics malignant perihilar cholangiocarcinoma, posing significant diagnostic challenges. This case is notable for initially normal preoperative IgG4 levels, followed by profound postoperative elevation, with final confirmation through histopathology. It highlights the need for heightened clinical vigilance and multidisciplinary evaluation in patients with atypical biliary strictures. This report expands awareness of the variable presentations of IgG4-SC and offers practical insights for its differential diagnosis and management.

**Case summary:**

A 72-year-old Chinese man presented with persistent right upper abdominal pain and acute-onset jaundice. Initial imaging and laboratory findings indicated severe cholestasis and suggested perihilar cholangiocarcinoma. Extensive preoperative imaging, percutaneous drainage, and surgical exploration were performed. Postoperative pathology revealed marked plasma cell infiltration and storiform fibrosis in the bile duct wall, with immunohistochemistry confirming abundant IgG4-positive plasma cells and a marked postoperative serum IgG4 increase (>1,600 mg/dL). The final diagnosis was IgG4-SC. Initiation of corticosteroid therapy led to rapid clinical, biochemical, and radiological improvement, with no adverse events and sustained remission at 9 months of follow-up.

**Conclusion:**

Definitive diagnosis of IgG4-SC requires integration of histopathology, not solely serological markers, to avoid misdiagnosis.

## Introduction

IgG4-SC is a rare autoimmune biliary disorder characterized by elevated serum IgG4 levels, extensive infiltration of IgG4-positive plasma cells, and progressive biliary tract fibrosis (1–3). As a distinct biliary manifestation of the broader spectrum of IgG4-related disease, IgG4-SC frequently presents with clinical, radiological, and laboratory features that closely mimic malignant perihilar cholangiocarcinoma and primary sclerosing cholangitis, often resulting in misdiagnosis and delayed intervention (4, 5). The pathogenesis of IgG4-SC remains incompletely understood, with current evidence suggesting complex interactions between autoimmune mechanisms, genetic susceptibility, and environmental triggers (2). Histologically, the disease is defined by dense lymphoplasmacytic infiltrates rich in IgG4-positive cells, obliterative phlebitis, and storiform fibrosis. Clinically, it typically presents non-specifically as jaundice, abdominal pain, and cholestatic liver dysfunction (6–9).

Despite advances in imaging and serological assessment, the definitive diagnosis of IgG4-SC relies upon a combination of histopathological examination and immunohistochemical confirmation (10). Importantly, serum IgG4 concentrations may be within normal limits in early or localized disease, and a single negative result does not exclude the diagnosis. Consequently, distinguishing IgG4-SC from malignant and other benign biliary strictures remains a major diagnostic challenge, with significant therapeutic implications (11–15). While IgG4-SC generally demonstrates a favorable response to corticosteroid-based immunosuppression, prompt and accurate differentiation from malignancy is essential to preventing unnecessary surgical intervention and optimize patient outcomes. To address these diagnostic complexities, we conducted a comprehensive literature review using search terms such as “IgG4-related sclerosing cholangitis,” “biliary stricture,” “cholangiocarcinoma mimic,” and “immunohistochemistry,” with a focus on recent advances in diagnosis and management, as well as reported case series highlighting clinical variability and diagnostic pitfalls (**Table 1**).

**Table 1:** Summary of case reports of IgG4-SC: diagnosis, treatment, and outcomes.

Reference	Case description	Diagnosis	Treatment	Outcome	Comparative insights
Shrestha P,2018 (22)	81-year-old man with yellowing of the skin and urine for 2 weeks; immunohistochemistry diagnosis: IgG4-SC.	IgG4-SC	Prednisone 40 mg, rituximab therapy, ERCP stenting.	Jaundice and liver function improved, with no recurrence observed during 2 years of follow-up.	Similar in elderly male presentation and histopathological confirmation. Differs in that preoperative IgG4 was normal in our case, rising dramatically postoperatively, and rituximab was not used.
Gyawali S,2023 (19)	An 83-year-old man presented with obstructive jaundice. Imaging suggested cholangiocarcinoma; however, CA19–9 was normal and IgG4 was elevated.	IgG4-SC	Oral corticosteroids (40 mg/day), gradually tapered and discontinued.	Clinical symptoms improved significantly within 1 month. No symptom recurrence was observed during 3 years of follow-up, with imaging and liver function remaining normal.	Both cases presented with obstructive jaundice and imaging mimicking malignancy. Unlike our case, preoperative IgG4 was already elevated, highlighting the variable serological timing in IgG4-SC.
Mittelstaedt A,2018 (20)	A 60-year-old man presented with painless jaundice. Imaging strongly suggested a Klatskin tumor, and CA19–9 was elevated.	IgG4-SC	Hilar resection with right hepatectomy.	Multiple postoperative complications occurred, and the patient died of acute respiratory failure 3 months after surgery.	Illustrates the risk of misdiagnosis leading to radical surgery. Our case underscores the importance of preoperative biopsy to avoid unnecessary resection, given similar imaging presentations.
Xu C, 2020 (23)	A 53-year-old man presented with jaundice for 5 months. Imaging suggested cholangiocarcinoma.	IgG4-SC	Corticosteroids (40 mg/day), gradually tapered.	Symptoms improved after corticosteroid therapy, with no recurrence during follow-up.	Shares the diagnostic challenge of malignancy mimicry and favorable steroid response. Our case uniquely demonstrates delayed IgG4 elevation post surgery, emphasizing the need for histology even with normal serology.
Roocroft H,2019 (21)	A 20-year-old man presented with acute abdominal pain as the initial symptom. Magnetic resonance cholangiopancreatography (MRCP) was performed to rule out common bile duct stones, as there was no jaundice.	IgG4-SC	Oral prednisone 40 mg/day; symptoms resolved after 2 weeks, and the dose was tapered to 10 mg/day for maintenance therapy after 4 weeks.	After 1 month of treatment, abdominal pain disappeared, and both liver function and serum IgG4 levels returned to normal. At 6 months, MRCP showed significant improvement of biliary and pancreatic lesions. No recurrence was observed during 1 year of follow-up.	Highlights atypical presentation without jaundice. Contrasts with our elderly jaundiced case, yet both responded promptly to steroids, supporting IgG4-SC’s broad clinical spectrum and treatment responsiveness.

This table summarizes multiple case reports of IgG4-SC, focusing on patient background, diagnostic process, treatment modalities, and outcomes. Treatment approaches included medical therapy (such as prednisone and rituximab), surgical intervention, and endoscopic procedures. The therapeutic effects and prognosis varied among cases: Most patients experienced symptom improvement with no recurrence during follow-up, whereas some developed postoperative complications leading to death. These findings reflect the complexity of treating IgG4-SC and highlight the importance of early diagnosis and long-term follow-up for prognosis.

Here, we present the case of a 72-year-old Chinese man who was initially suspected of having perihilar cholangiocarcinoma based on clinical and imaging findings, but was ultimately diagnosed with IgG4-SC following surgical intervention and pathological analysis. This case underscores the unique diagnostic dilemmas posed by IgG4-SC, particularly the potential for normal preoperative IgG4 levels and the critical value of histopathology in establishing the correct diagnosis. By integrating clinical, biochemical, radiological, and pathological perspectives, this report contributes to the growing body of literature advocating for increased clinical vigilance and multidisciplinary evaluation in patients with atypical biliary strictures and offers practical guidance for the differential diagnosis and management of this challenging clinical entity.

## Case presentation

A 72-year-old Chinese man, height and weight not recorded, retired, presented to the hospital on March 22, 2025, with a chief complaint of persistent dull pain in the right upper abdomen for 1 week and new-onset jaundice for 1 day. The abdominal pain was continuous, non-radiating, and without apparent precipitating factors. Three days prior to admission, the patient noticed yellow discoloration of the skin and sclera accompanied by darkened urine. He denied fever, chills, nausea, or vomiting. There were no reports of recent weight loss, change in appetite, or night sweats.

The patient’s medical history was significant for longstanding hypertension, for which he had not been on regular antihypertensive therapy. He also had a history of chronic bronchitis with emphysema, but there was no prior diagnosis or symptoms suggestive of hepatitis, tuberculosis, or other infectious diseases. His surgical history included an appendectomy and a prior excision of a postauricular lymph node. There was no history of diabetes mellitus, autoimmune disorders, or malignancy. Regarding family history, there was no known history of hepatic, biliary, or autoimmune diseases among immediate relatives. The patient reported no history of tobacco, alcohol, or illicit drug use. He had not traveled recently, nor was there exposure to environmental toxins or known hepatobiliary carcinogens. Psychosocial history was unremarkable, and there were no significant occupational exposures.

On admission, the patient’s medication history was carefully reviewed. He reported no current use of prescription medications, over-the-counter drugs, herbal preparations, or supplements. He had not received recent vaccinations or depot injections. Adherence to prior antihypertensive prescriptions was poor, and no medication allergies were documented. There was no history of adverse drug reactions, and the patient’s drug allergy status was negative for both generic and branded drugs.

Dietary history revealed a typical Chinese diet, with no history of excessive alcohol intake or recent changes in nutrition. There was no report of parenteral nutrition or restricted diets prior to admission. The patient had not experienced any recent dietary intolerances.

Upon physical examination, the patient was alert and oriented, with vital signs within normal limits. He appeared icteric, but there was no evident distress. The abdominal exam revealed a flat abdomen with mild tenderness in the right upper quadrant. No rebound tenderness or muscle rigidity was noted, and Murphy’s sign was negative. There was no palpable hepatosplenomegaly, no shifting dullness, and no peripheral edema. Cardiopulmonary and neurological examinations showed unremarkable results.

Preoperative clinical, laboratory, and imaging findings (March 22–April 2, 2025).

During the initial hospitalization from March 22 to March 28, 2025, serial laboratory evaluations were performed. Preoperative liver function tests demonstrated significant elevations in alanine aminotransferase, aspartate aminotransferase, total bilirubin (261.2 μmol/L), and direct bilirubin (158.1 μmol/L), indicative of severe cholestasis and hepatocellular injury. Alkaline phosphatase (ALP) and gamma-glutamyl transpeptidase (gGT) were markedly increased to 1,245 and 587 U/L, respectively, consistent with obstructive jaundice. Renal function and serum electrolytes were within reference ranges, as were hematological indices. Urinalysis revealed dark urine, but was otherwise unremarkable. Fecal occult blood showed a negative result. Blood biochemistry showed no abnormalities in glucose, cholesterol, or triglycerides. Immune indices were notable for a serum IgG of 18.4 g/L measured on March 27; serum IgG4 was not tested preoperatively. Infection markers, including C-reactive protein, were elevated during the acute phase. There was no eosinophilia or serological evidence of active hepatitis (**Table 2**).

**Table 2:** Changes in liver function and IgG levels in patients.

Date	Days from admission	Days from surgery	ALT (U/L)	AST (U/L)	TBIL (μmol/L)	DBIL (μmol/L)	IgG (g/L)	IgG4 (mg/dL)
2025/3/22	Day 1	–	267	143	261.2	158.1	–	–
2025/3/27	Day 6	–	–	–	–	–	18.4	–
2025/3/28	Day 7	–	197	165	153.4	101.2	–	–
2025/4/3	Day 13	Post-op day 0 (surgery performed)	–	–	–	–	–	–
2025/4/11	Day 21	Post-op day 8	252	357	43.8	31.5	–	–
2025/4/17	Day 27	Post-op day 14	223	175	46.5	28.3	–	–
2025/5/10	Day 50	Post-op day 37	–	–	–	–	27.2	>1600
2025/5/14	Day 54	Post-op day 41	137	63	38.8	24.1	–	–
2025/5/16	Day 56	Post-op day 43	81	31	31.2	20.4	–	–

ALT, alanine aminotransferase; AST, aspartate aminotransferase; TBIL, total bilirubin; DBIL, direct bilirubin; IgG, immunoglobulin G; IgG4, immunoglobulin G4.

This table displays the dynamic changes in liver function indicators (ALT, AST, TBIL, DBIL) and immunoglobulin levels (IgG, IgG4) during the period from March 22 to May 16,2025. It records specific values of each indicator at different time points (day 1 to day 56), with untested indicators indicated by “-”.

**Figure 1:**
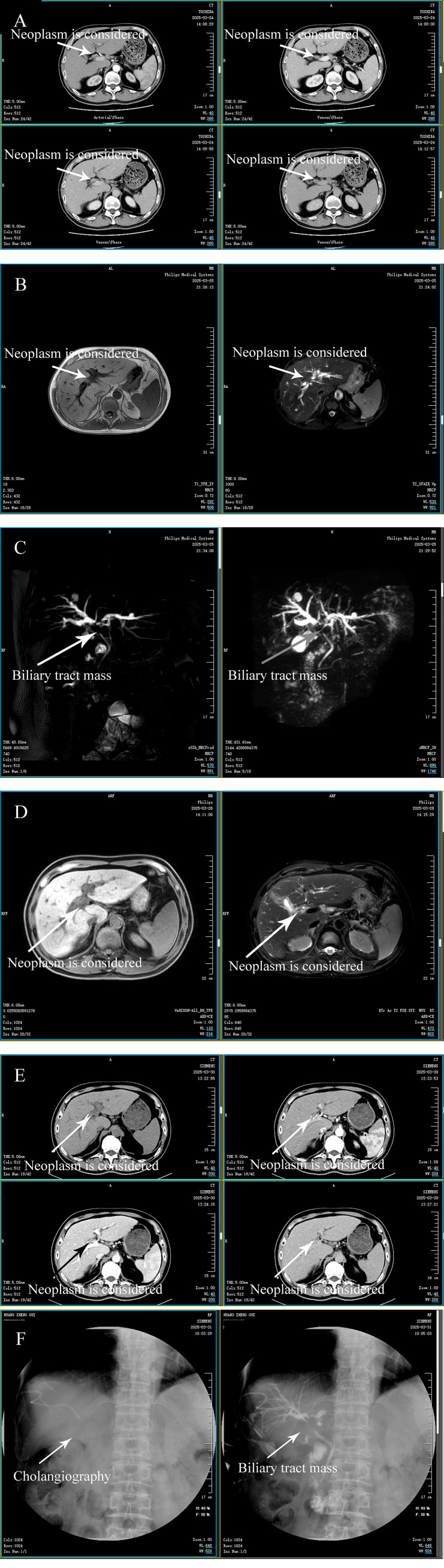
Imaging findings of IGG4-related cholangitis. **(A, B)** Plain and enhanced CT examinations of the liver, gallbladder, spleen, and pancreas, with neoplasms indicated. **(C, D)** MRCP (magnetic resonance cholangiopancreatography) of the pancreaticobiliary system, showing a biliary tract mass. **(E, F)** Plain and enhanced MR examinations of the liver, with neoplasms indicated.

Imaging studies played a critical role in the preoperative diagnostic evaluation. Initial abdominal ultrasound revealed marked intrahepatic biliary dilation, gallbladder enlargement with wall thickening and sludge, and concentric thickening of the hilar bile duct with luminal narrowing, findings suggestive of perihilar cholangiocarcinoma. Subsequent contrast-enhanced magnetic resonance imaging (MRI) confirmed significant intrahepatic bile duct dilatation and mural thickening of the common hepatic duct and confluence. It also revealed multiple enlarged lymph nodes in the hepatic hilum and retroperitoneum, gallstones, and inflammatory changes in the gallbladder. Mild atrophy of the pancreatic body and haziness of peripancreatic fat indicated possible chronic pancreatitis. Computed tomography angiography (CTA) and venography corroborated these findings, with no evidence of vascular invasion. Percutaneous transhepatic cholangial drainage (PTCD) was performed preoperatively, providing temporary relief of biliary obstruction, although cholangiography demonstrated poor visualization of the hilar bile ducts due to severe obstruction. Chest computed tomography revealed no evidence of pulmonary involvement, and brain imaging was not performed due to the absence of neurological symptoms. Detailed imaging findings are provided in **Figure 1**, which collectively illustrate marked intrahepatic biliary dilation, concentric hilar bile duct thickening, and peripancreatic inflammatory changes consistent with IgG4-SC.

## Surgical intervention and intraoperative findings (April 3, 2025)

Surgical intervention was undertaken on April 3, 2025, consisting of cholecystectomy, open exploration, hilar lymphadenectomy, and bile duct core needle biopsy. Intraoperative findings were consistent with preoperative imaging: The liver was enlarged and firm, the gallbladder was distended with a thickened wall and adhered to surrounding tissue, and there was mild peritoneal fluid. No overt neoplastic lesion or lymphatic metastasis was identified in the hepatic hilum. Bile duct biopsy specimens were obtained from the sclerotic confluence of the left and right hepatic ducts.

## Postoperative pathological, laboratory, and clinical course (April 3, 2025 onward)

**Figure 2:**
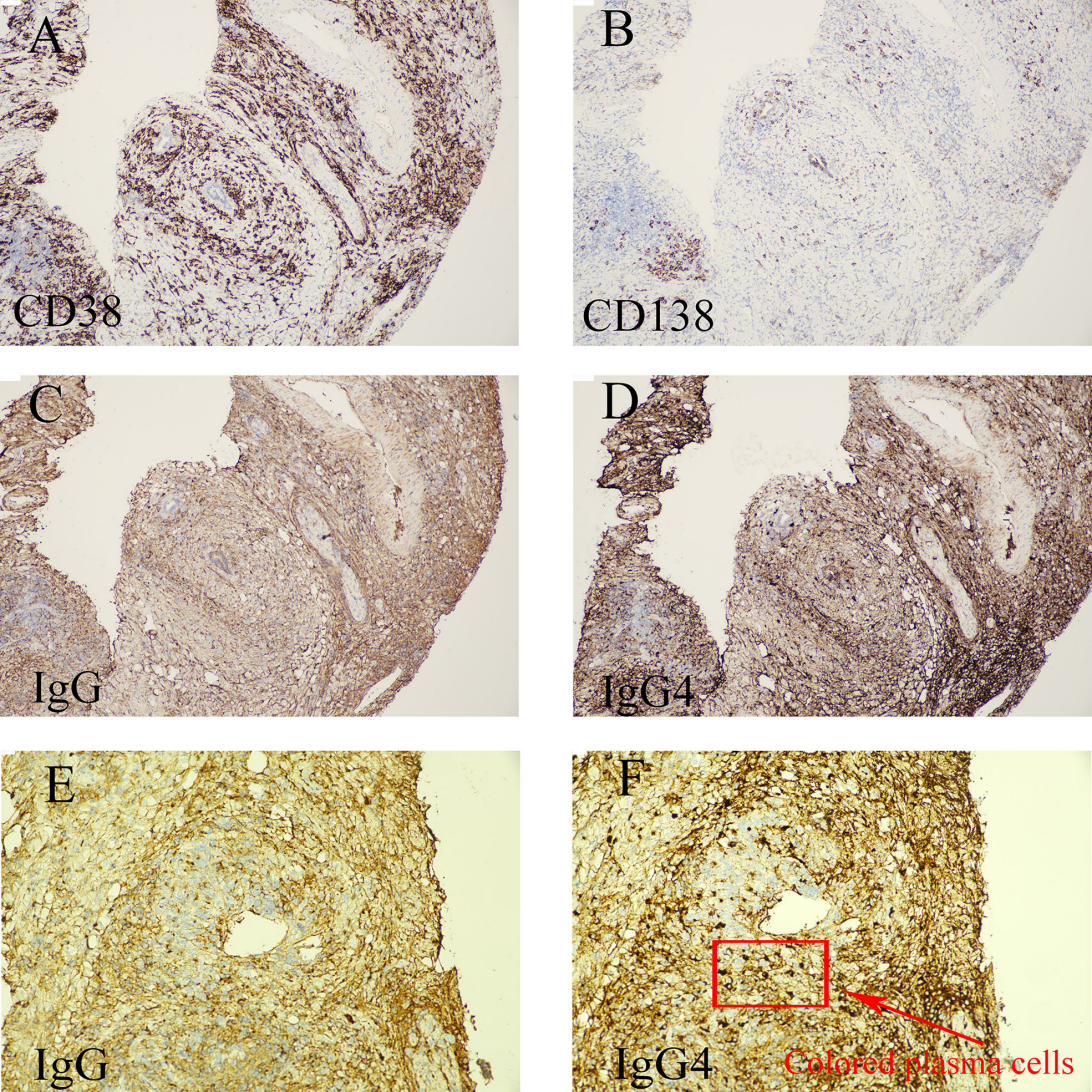
(Common bile duct needle biopsy specimen). **(A–D)** (low-power magnification) demonstrate the overall distribution of immunoreactive cells. Immunohistochemistry shows scattered IgG4-positive plasma cells within the biopsy specimen. Overall, these findings support the possibility of IgG4-related sclerosing cholangitis (IgG4-SC); however, given the limited sampling inherent to needle biopsy, clinicoradiologic correlation is recommended, including serum IgG4 measurement. Immunohistochemical profile: PCK (+); CD38 (positive in plasma cells and lymphocytes); CD138 (positive in plasma cells); IgG4 (scattered positive); IgG (focal/occasional positive). In this specimen, IgG4-positive plasma cells appear to outnumber IgG-positive plasma cells. **(E, F)** (high-power magnification): Panel E (IgG) shows prominent non-specific/background staining, with only rare plasma cells exhibiting strong cytoplasmic positivity. **(F)** (IgG4) shows more than 30 strongly IgG4-positive plasma cells per high-power field, displaying a periductal and perivenular distribution with infiltration around small-caliber venules.

Postoperative pathology revealed chronic cholecystitis with cholesterolosis and numerous sand-like gallstones, with the gallbladder wall measuring up to 0.3 cm in thickness. Nine resected lymph nodes showed reactive hyperplasia without malignancy. Critically, histology of the common bile duct showed marked plasma cell infiltration and fibrosis surrounding small bile ducts, with prominent intrahepatocytic cholestasis. Immunohistochemistry demonstrated scattered IgG4-positive plasma cells, exceeding the number of IgG-positive cells. Histopathological examination of the bile duct biopsy revealed a dense lymphoplasmacytic infiltrate accompanied by storiform fibrosis. Immunohistochemical quantification demonstrated a ratio of IgG4-positive to IgG-positive plasma cells exceeding 40%, with IgG4+ plasma cells numbering >50 per high-power field, thereby fulfilling the major histological criteria for IgG4-SC. Detailed pathological findings are provided in **Figure 2**, demonstrating dense lymphoplasmacytic infiltrates with storiform fibrosis and a predominance of IgG4-positive plasma cells, key histological features supportive of IgG4-SC.

Postoperative laboratory monitoring revealed a marked elevation in serum immunoglobulin levels. By May 10, 2025 (postoperative day 37), serum IgG had risen to 27.20 g/L, and serum IgG4 was dramatically elevated to >1,600 mg/dL (reference range < 135 mg/dL) (**Table 2**). Concurrently, the patient’s hepatic enzymes and bilirubin levels gradually declined following surgery and with subsequent medical therapy.

Following surgery, the patient was started on oral corticosteroid therapy (prednisone, initial dose 30–40 mg/day), with a gradual tapering protocol. Repeat imaging revealed significant resolution of intrahepatic biliary dilation, reduction of peritoneal and pleural effusions, and decreased lymphadenopathy. The PTCD tube was removed approximately 1 month postoperatively. Although thickening of the common bile duct wall persisted, the overall radiographic appearance improved significantly.

At 9 months postoperatively, the patient’s clinical status remained stable, with sustained normalization of liver biochemistry and IgG4 levels, and near-complete resolution of radiological abnormalities (**Table 2**). The surgical wound had healed well, and no adverse events were recorded. Adherence to the prescribed treatment regimen was confirmed through regular outpatient visits and laboratory follow-up.

## Discussion

The present case report of IgG4-SC in a 72-year-old Chinese man illustrates the significant diagnostic and therapeutic challenges posed by this rare autoimmune biliary disorder, particularly given its substantial clinical and radiological overlap with perihilar cholangiocarcinoma and primary sclerosing cholangitis. In the literature, IgG4-SC is characterized by elevated serum IgG4 levels, extensive infiltration of IgG4-positive plasma cells, and variable degrees of biliary tract fibrosis. However, it often masquerades as malignancy, leading to frequent misdiagnosis and inappropriate management (1, 2, 16). Our findings are concordant with prior reports emphasizing that, despite advances in imaging and serology, definitive diagnosis remains highly reliant on histopathology and immunohistochemistry. Furthermore, the present case meets the 2019 ACR/EULAR classification criteria for IgG4-RD, as evidenced by characteristic histopathology (dense lymphoplasmacytic infiltrate with >50 IgG4+ plasma cells and storiform fibrosis), elevated serum IgG4 (>1,600 mg/dL), and typical imaging features of biliary involvement.

Comparative analysis between the present case and published series reveals both commonalities and distinctions. Like the majority of reported IgG4-SC cases, this patient initially presented with obstructive jaundice, abdominal pain, and abnormal liver biochemistry suggestive of cholestasis and hepatocellular injury (17). Imaging modalities—including ultrasound, MRI, and CTA—demonstrated intrahepatic biliary dilation, bile duct wall thickening, and lymphadenopathy, findings which have been widely documented as hallmarks of both IgG4-SC and cholangiocarcinoma (5, 16, 18). However, the marked postoperative elevation of serum IgG4 (>1,600 mg/dL), together with immunohistochemical evidence of dense IgG4-positive plasma cell infiltration in the bile duct wall, was critical for confirming the diagnosis of IgG4-SC, consistent with established histological criteria (11). Demographically, this case aligns with the typical profile of IgG4-SC patients reported in the literature, who are predominantly elderly men. As summarized in **Table 1**, the majority of reported cases occur in men over 60 years of age, with a peak incidence in the seventh to eighth decades (19–23).Our patient, a 72-year-old man, fits this pattern. However, variations exist: While most cases present with obstructive jaundice, some younger patients (e.g., the 20-year-old man reported by (21)) may present atypically without jaundice, underscoring the clinical heterogeneity of IgG4-SC. Comorbidities in IgG4-SC are often non-specific; our patient had hypertension and chronic bronchitis with emphysema, which are common in the elderly population but not directly linked to IgG4-SC pathogenesis. In contrast, some reported cases note concomitant autoimmune pancreatitis or other IgG4-related organ involvement, highlighting the systemic nature of the disease. These demographic and clinical parallels reinforce that IgG4-SC should be considered in elderly men presenting with biliary strictures, even in the absence of typical autoimmune comorbidities.

Notably, this case highlights an important nuance: Preoperative IgG4 was not measured; however, postoperative IgG4 was markedly elevated (>1,600 mg/dL). This temporal dissociation, although reported in the literature, underscores the limitation of relying on a single preoperative serological test and supports the argument for serial measurement and the integration of histopathological findings. The sharp postoperative rise in IgG4 may be attributed to surgical stress and enhanced immune activation following intervention. Similar observations have been reported in IgG4-related inflammatory abdominal aortic aneurysm, where endovascular repair (EVAR) was associated with significant postoperative increases in serum IgG4 levels and progression of periaortic fibrosis compared with open surgery, suggesting that procedural trauma may exacerbate the underlying IgG4-related immune response (24). Such cases reinforce the growing recognition that normal serum IgG4 does not exclude IgG4-SC, especially in early or localized disease, as discussed in recent reviews and consensus guidelines (25).

Causal inference is strengthened by the typical pathological features observed in the surgical specimen: prominent periductal fibrosis, chronic inflammatory infiltrate rich in IgG4-positive plasma cells, and absence of neoplastic tissue. The clinical course further supports causality; the patient’s rapid biochemical and radiological improvement following corticosteroid therapy is consistent with IgG4-SC rather than malignancy, as the literature repeatedly describes robust steroid responsiveness as a distinguishing feature of IgG4-SC (26).

Temporality is evident from the sequential development of symptoms, laboratory abnormalities, and imaging findings, followed by diagnostic clarification through surgical intervention and subsequent clinical remission upon initiation of immunosuppression. This chronology affirms the diagnosis and aligns with the natural history of IgG4-SC, as delineated in longitudinal case series (27).

The diagnosis of IgG4-SC in this case is further corroborated by its alignment with established histopathological criteria for IgG4-related disease. As outlined in the international consensus statement on IgG4-related disease pathology, definitive diagnosis relies on characteristic histological features including dense lymphoplasmacytic infiltrates, storiform fibrosis, obliterative phlebitis, and an elevated IgG4+/IgG+ plasma cell ratio (>40%) or >50 IgG4+ plasma cells per high-power field. The present case fulfills these criteria, demonstrating marked IgG4-positive plasma cell infiltration (>50/hpf) and storiform fibrosis in bile duct biopsy specimens. This histopathological confirmation, together with the typical imaging findings and dramatic response to corticosteroid therapy, strongly supports the diagnosis of IgG4-SC, consistent with established diagnostic frameworks (6).

Despite the robust clinical and histopathological evidence, several limitations must be acknowledged. First, the absence of preoperative tissue diagnosis and reliance on postoperative biopsy could have introduced diagnostic delay (28). Second, serum IgG4 levels were not serially measured prior to intervention, limiting the evaluation of their dynamic change. Third, as a single case report, the generalizability is inherently limited, and the absence of long-term follow-up precludes assessment of recurrence or delayed complications. Furthermore, as noted in the literature, variations in the technical quality of biopsy sampling and immunohistochemistry may lead to underdiagnosis or misclassification (29). Potential variation in clinical outcomes also arises from patient-specific factors such as age, comorbidities, and individual immune response, as well as from institutional differences in diagnostic and therapeutic protocols (30).

From a methodological perspective, this case underscores the need for multidisciplinary collaboration and systematic follow-up, as well as the integration of clinical, biochemical, radiological, and pathological data for accurate diagnosis. The literature supports early and aggressive use of corticosteroids but also highlights the risk of relapse and the potential necessity for adjunctive immunosuppressive therapy, particularly in patients with multiorgan involvement or steroid-refractory disease (31). However, access to histopathology and advanced imaging may be limited in some settings, potentially impeding optimal management.

The uniqueness of this case lies in its initial clinical mimicry of cholangiocarcinoma, the delayed yet profound elevation in serum IgG4, and the demonstration of dramatic clinical improvement with corticosteroid therapy. This constellation of features, supported by extensive literature, validates the diagnosis and reinforces the critical importance of considering IgG4-SC in the differential diagnosis of obstructive jaundice, especially in elderly men with biliary strictures and atypical imaging findings (17, 32).

## Conclusion

In conclusion, this case highlights the diagnostic complexity of IgG4-SC, which frequently mimics malignant biliary disease and may be underrecognized in the absence of elevated serum IgG4. The successful outcome in this patient, marked by clinical, biochemical, and radiological remission following corticosteroid therapy, underscores the necessity of integrating histopathological and immunohistochemical assessment—rather than relying solely on serological markers—for definitive diagnosis and management. Evidence from this case and the literature supports early initiation of steroid-based therapy, with adjunctive immunosuppressants considered in refractory or relapsing cases. Clinicians should maintain a high index of suspicion for IgG4-SC in patients with biliary strictures and cholestatic liver dysfunction, even when initial serological results are inconclusive, and should pursue tissue diagnosis when feasible. The application of these diagnostic and therapeutic principles in clinical practice has the potential to prevent misdiagnosis, guide individualized treatment, and improve patient outcomes. Further research is warranted to clarify the pathogenesis of IgG4-SC, establish optimal strategies for long-term management, and identify biomarkers for earlier and more accurate diagnosis.

## Data Availability

The original contributions presented in the study are included in the article/supplementary material. Further inquiries can be directed to the corresponding authors.
